# Magnetic hydrogel particles improve nanopore sequencing of SARS-CoV-2 and other respiratory viruses

**DOI:** 10.1038/s41598-023-29206-7

**Published:** 2023-02-07

**Authors:** P. Andersen, S. Barksdale, R.A. Barclay, N. Smith, J. Fernandes, K. Besse, D. Goldfarb, R. Barbero, R. Dunlap, T. Jones-Roe, R. Kelly, S. Miao, C. Ruhunusiri, A. Munns, S. Mosavi, L. Sanson, D. Munns, S. Sahoo, O. Swahn, K. Hull, D. White, K. Kolb, F. Noroozi, J. Seelam, A. Patnaik, B. Lepene

**Affiliations:** Present Address: Ceres Nanosciences, Inc., Manassas, VA 20110 USA

**Keywords:** Sequencing, Biotechnology, Computational biology and bioinformatics, Molecular biology, Biomarkers, Health care, Molecular medicine, Biological techniques, High-throughput screening

## Abstract

Presented here is a magnetic hydrogel particle enabled workflow for capturing and concentrating SARS-CoV-2 from diagnostic remnant swab samples that significantly improves sequencing results using the Oxford Nanopore Technologies MinION sequencing platform. Our approach utilizes a novel affinity-based magnetic hydrogel particle, circumventing low input sample volumes and allowing for both rapid manual and automated high throughput workflows that are compatible with Nanopore sequencing. This approach enhances standard RNA extraction protocols, providing up to 40 × improvements in viral mapped reads, and improves sequencing coverage by 20–80% from lower titer diagnostic remnant samples. Furthermore, we demonstrate that this approach works for contrived influenza virus and respiratory syncytial virus samples, suggesting that it can be used to identify and improve sequencing results of multiple viruses in VTM samples. These methods can be performed manually or on a KingFisher automation platform.

## Introduction

As of April 10, 2022, there have been more than 500 million COVID-19 cases and nearly 6.2 million COVID-19 related deaths worldwide^1^. Viral mutations have enabled the pandemic to pervade everyday life despite the use of widespread global health measures to prevent the spread of SARS-CoV-2. Detection and monitoring of emerging viral variants have become critical tools in the global health response, highlighting the need for rapidly deployable and accurate sequencing methods^2–5^. Recent advances in next-generation sequencing (NGS) technologies have made the routine use of sequencing for monitoring and identifying viral outbreaks more possible, but many NGS instruments are not portable and still cost prohibitive, thus limiting their overall adoption^6,7^. The Oxford Nanopore Technologies (ONT) MinION platform offers a relatively inexpensive and portable detection strategy, one that is capable of identifying and sequencing various respiratory viruses in the field^8–10^.

While sequencing advances make rapid on-site detection and characterization of SARS-CoV-2 and other viral genomes a possibility, these portable sequencers are still limited by certain disadvantages, namely that unless large amounts of viral RNA are used for the sequencing reactions, there can be accuracy issues during basecalling^11–16^. These technical limitations reduce the usefulness of a tool that could improve the ability to rapidly and accurately respond to viral outbreaks and track transmission in real time.

Increasing the total amount of RNA material for analysis through sample enrichment is one strategy available for improving the performance of sequencing platforms. To this end, we sought to address the viral sequencing limitations of a nanopore sequencer by applying the affinity-based magnetic hydrogel particle (Nanotrap particle) enrichment technology to SARS-CoV-2 viral transport medium (VTM) samples. Briefly, magnetic hydrogel particles improve assay performance by facilitating rapid analyte (e.g. intact virions) binding from large sample volumes and by reducing the presence of interfering substances in downstream assays. The hydrogel particles capture and concentrate analytes of interest using small molecules, like affinity dyes, that are immobilized on a framework of crosslinked polymer chains. These small molecules bind to their targets (e.g. virion surface proteins), often with very high affinity, through a combination of electrostatic and hydrophic interactions. Once bound to the magnetic hydrogel particle, the virions can be concentrated and removed from the sample matrix using a simple magnetic separation step. After concentration, the virions are tightly bound to the hydrogel particle affinity dye and the viral nucleic acids are not available for molecular analysis. As such, a nucleic acid extraction kit is used to lyse the virions and purify the viral nucleic acids in preparation for a downstream molecular assay. The Nanotrap particle technology has shown broad application in clinical diagnostics by enriching and stabilizing biomarkers and analytes in complex clinical samples. Recent studies demonstrated this concentration and extraction process, showing that the magnetic hydrogel particles are able to concentrate and improve detection of many viral types, including SARS-CoV-2, on multiple molecular assays^17–21^.

When utilizing a portable sequencing platform such as the ONT MinION sequencer, increasing the amount of input RNA should enable successful sequencing of lower titer viral samples, which, in turn, potentially increases the fraction of patient samples that are viable for sequencing. Ideally, this should improve identification of specific viral mutations, hastening the response to emerging problematic variants^22–24^.

Here, we show that magnetic hydrogel particles (Nanotrap particles) can improve current sequencing workflows and enable new ones by enhancing current standard RNA extraction methods. We demonstrate that these magnetic hydrogel particle (Nanotrap particle) workflows improve sequencing results by increasing total viral mapped reads, resulting in greater sequencing depth and coverage. Nanotrap particle workflows were developed for multiple RNA extraction kits, and their utility is demonstrated in both contrived and diagnostic remnant samples.

## Methods and materials

### Magnetic hydrogel particles

Nanotrap Magnetic Virus Particles (SKU: 44,202) were provided by Ceres Nanosciences Inc. Manassas, VA.

### Biological materials

Contrived samples were comprised of heat-inactivated virus spiked into VTM (Puritan UniTranz-RT Transport Systems, cat# 89,233–458). 5 concentrations were generated (10^6^ ,10^5^, 10^4^, 10^3^, and 10^2^ TCID_50_/mL) starting with a neat VTM sample spiked at 10^6^ TCID_50_/mL from the viral stock, four 1:10 serial dilutions were performed down to a concentration of 10^2^ TCID_50_/mL using neat VTM as the dilution buffer. Starting viral stock concentration was based on the manufacturer’s reported concentration. Heat-inactivated viruses were purchased from ZeptoMetrix: SARS-CoV-2 (cat# 0810587CFHI), Influenza A-H1N1 (cat# 0810109CFHI), and Respiratory Syncytial Virus Type A(RSV) (cat# 0810040ACFHI).

### Clinical samples

SARS-CoV-2 positive diagnostic remnant samples in viral transport medium were purchased from Discovery Life Sciences, Huntsville, AL. Discovery Life Sciences previously tested these samples by RT-PCR, obtaining cycle thresholds ranging from 24 to 35. Samples acquired from Discovery Life Sciences follow the “Biospecimen Purchase Agreement” and are de-identified with the following data generation adhering to their identification guidelines, allowing for the use of such samples for research and publication, including qPCR and sequencing.

### Magnetic hydrogel particle workflows

*Nanotrap Particle Workflow 1* (Manual Magnetic Hydrogel Particle Method with Column-Based RNA Extraction Kit): Two hundred microliters of magnetic hydrogel particles (Nanotrap particles) at a concentration of 5 mg/mL were added to 1000 µL VTM samples. The amount of Nanotrap particles utilized was based upon previous virus capture optimization experiments^17–21^. Samples were incubated at room temperature for 10 min, and then placed on a magnetic separator for 2 min to allow the Nanotrap particles to pellet. Supernatants were removed and discarded. One hundred microliters of RNAse/DNase-free water with 350 µL of QIAGEN Buffer RLT were added to the Nanotrap particle pellet. Samples were incubated for 10 min on a shaker at room temperature before being placed on a magnetic separator for 2 min to allow the Nanotrap particles to pellet. Supernatants containing the viral nucleic acid material were processed for RNA extraction using the QIAGEN RNeasy MinElute Cleanup Kit (cat #74,204) following the manufacturer’s 100 µL workflow instructions. Following RNA extraction, RNA samples were ready for sequencing library preparation.

*Nanotrap Particle Workflow 2* (Manual Magnetic Hydrogel Particle Method with Magnetic Bead-Based RNA Extraction Kit): Three hundred microliters of PBS with 0.05% Tween-20 (v/v) was added directly to 500 µL VTM samples. Two hundred microliters of magnetic hydrogel particle (Nanotrap particles) were added to each VTM sample, which were then incubated at room temperature for 10 min. The amount of Nanotrap particles utilized for these experiments was based upon previous successful virus capture workflows and was optimized for performance and cost^17–21^. Samples were placed on a magnetic separator for 2 min to allow the Nanotrap particles to pellet. Supernatants were removed and discarded. Nanotrap particle pellets were resuspended in 1 mL of molecular grade water with 0.05% Tween-20 (v/v). Following a brief resuspension, the samples were again placed on a magnetic separator for 2 min to allow the Nanotrap particles to pellet, and the supernatant removed and discarded. Nanotrap particles were resuspended in 200 µL of MagMAX Microbiome Lysis Solution, and samples were incubated at 65 °C on a shaker for 10 min. Samples were then placed on a magnetic separator to allow the Nanotrap particles to pellet for 2 min. Supernatants containing the viral nucleic acid material underwent RNA extraction using the using the MagMAX Microbiome Ultra Nucleic Acid Isolation Kit (cat# A42357) following the manufacturer’s 200 µL workflow instructions. Following extraction kit processing, RNA samples were ready for sequencing library preparation.

*Nanotrap Particle Workflow 3* (Automated Magnetic Hydrogel Particle Method with Magnetic Bead-Based RNA Extraction Kit): The following method used the KingFisher automation platform (KingFisher Apex) and associated consumables. Three hundred microliters of PBS with 0.05% Tween-20(v/v) was added directly to 500 µL VTM samples in a 96 deep well KingFisher plate. Two hundred microliters of magnetic hydrogel particles (Nanotrap Particles) were added to each VTM sample. The amount of Nanotrap particles utilized for these experiments was based upon previous successful virus capture workflows and was optimized for performance and cost^17–21^. Molecular grade water with 0.05% Tween-20 (v/v) was added to a second 96 DW plate, and 200 µL of MagMAX Microbiome Lysis Solution was added to a third 96 DW plate. A custom KingFisher program “NT2MM.kfx” was made to process the Nanotrap particles using the three prepared 96 DW plates. The entire process occurred in 30 min, and the final eluate contained extracted viral RNA.

After magnetic hydrogel particle (Nanotrap particle) processing, similar to “Nanotrap Particle Workflow 2”, extracted viral RNA samples were processed using the MagMAX Microbiome Ultra Nucleic Acid Isolation Kit following the manufacturer’s 200 µL workflow instructions. For this workflow the extraction was automated on the KingFisher automation platform. Nanotrap Particle Workflow 2 and 3 are considered by the authors to be functionally the same, using the same reagents and extraction conditions, with the only appreciable difference being that “Nanotrap Particle Workflow 3” is conducted on the KingFisher automation platform. Following extraction kit processing, RNA samples were ready for sequencing library preparation.

### RNA extraction kits

The magnetic hydrogel particle (Nanotrap particle) workflows described above were benchmarked against two standard extraction kit workflows without any Nanotrap particles. For Workflow 1 comparison, samples were processed using the QIAGEN RNeasy MinElute Cleanup Kit(cat #74,204) following the manufacturer’s 100 µL workflow instructions. For Workflow 2 and 3 comparisons, samples were processed using the ThermoFisher MagMAX Microbiome Ultra Nucleic Acid Isolation Kit (cat# A42357) following the manufacturer’s 200 µL workflow instructions.

### Enrichment factor

The magnetic hydrogel particle (Nanotrap particle) method enables a user to interrogate a larger sample input volume relative to other extraction methods. For Nanotrap Particle Workflow 1, the theoretical enrichment factor is 10 × as our method processes 1000 µL of VTM relative to the 100 µL input volume of the column manufacturer’s recommendations. Note that, given the limitations in the volumes of clinical VTM collections, we restricted the volumes used for Nanotrap Workflow 1 µL to 1000 µL.

For Nanotrap Particle Workflow 2 and 3, the theoretical enrichment factor is 2.5 × as our method processes 500 µL of VTM relative to the 200 µL input volume of the silica bead manufacturer’s recommendations. To allow for automation, we restricted the volumes used for Workflow 2 and 3, ensuring compatibility with the KingFisher automation platform.

### RT-PCR

For RT-PCR analysis of SARS-CoV-2 samples, the IDT 2019 nCoV CDC EUA Kit (cat# 1,006,770), which includes N1 primers/probes, was used for real-time RT-PCR. Following IDT’s recommendation, TaqPath 1-Step RT-qPCR Master Mix from ThermoFisher (cat# A15300) was used in the IDT 2019 nCoV CDC EUA assay. Each PCR reaction used 8.5 µL of nuclease free water, 5 µL of the TaqPath solution, 1.5 µL of the N1 primer/probe, and 5 µL of RNA template. PCR conditions were performed according to IDT’s instructions on a Roche LightCycler 96. All SARS-CoV-2 (both heat-inactivated and diagnostic remnant) experiments utilized this assay.

For RT-PCR analysis of Influenza A and RSV samples, the Primerdesign Influenza A H1 Kit (Path-H1N1-v2.0-Standard) and the Primerdesign RSV kit (Path-RSV-A-Standard) were used following the manufacturer’s instructions. PCR conditions were performed according to Primerdesign’s instructions on a Roche LightCycler 96.

### Library preparation and sequencing workflow

After viral RNA extraction, samples were prepared for sequencing using the ARTIC Network developed; “nCoV-2019 Sequencing Protocol v3(Lo Cost)”^25^. Briefly, amplified cDNA was prepared using a targeted amplicon approach. Per the ARTIC nCov-2019 protocol, IDT ARTIC V3 Amplicon Sequencing Panel primers were used. These 218 primers, covering the entire SARS-CoV-2 genome, were used to generate and amplify cDNA from the extracted viral RNA. Once cDNA was prepared, the samples were processed using the Oxford Nanopore Technologies (ONT) Ligation Sequencing Kit, barcoded individually using the ONT Native barcoding expansion kit native barcodes with a modified “One-pot” protocol following the “nCov-2019 Sequencing Protocol v3(Lo Cost)” instructions. These individual samples were pooled together and concentrated using AMPure XP magnetic beads (cat# A63880). The pooled library was loaded onto an ONT FLO-MIN106 R.9 flow cell used with the ONT Mk1C Sequencing Platform. Unless otherwise stated, the ONT Mk1C was run for 24 h using the LSK109 kit with EXP-ND-196 barcodes selected.

### Bioinformatics and data analysis

To analyze and process the sequencing data generated by the ONT Mk1C platform, the following tools were used: live basecalling and demultiplexing was performed using the ONT MinKnow software integrated into the ONT Mk1C MinION device; general classification and viral mapped reads were generated using the 3/9/2020 W.I.M.P protocol through ONT’s Epi2me web tool; further coverage analysis was conducted using Minimap2 and Samtools through the UseGalaxy.org web portal. Paired *t*-tests were performed and figures were generated using Graphpad Prism 9. Nextclade and Pangolin analysis was performed through the EPI2ME Labs developed ARTIC SARS-CoV-2 Workflow^26^.

## Results

Prior studies have demonstrated that magnetic hydrogel particles (Nanotrap particles) capture and concentrate multiple respiratory viral pathogens, including SARS-CoV-2, Influenza A, Influenza B, and RSV^17–21^. This enrichment led to improved results by various molecular assays, including real-time RT-PCR. We hypothesized that if the Nanotrap particles could improve these molecular assays, they could also improve sequencing platforms by increasing the amount of viral RNA available for sequencing. To demonstrate the robustness and ease-of-use of the Nanotrap particle technology in sequencing, three workflows were developed—a manual method with a column-based RNA extraction kit, a manual method with a magnetic-bead-based RNA extraction kit, and an automated method with a magnetic-bead-based RNA extraction kit.

### Magnetic hydrogel particles (nanotrap particles) improve ONT sequencing results for contrived SARS-CoV-2 VTM samples using a column-based RNA extraction method

**Figure 1:**
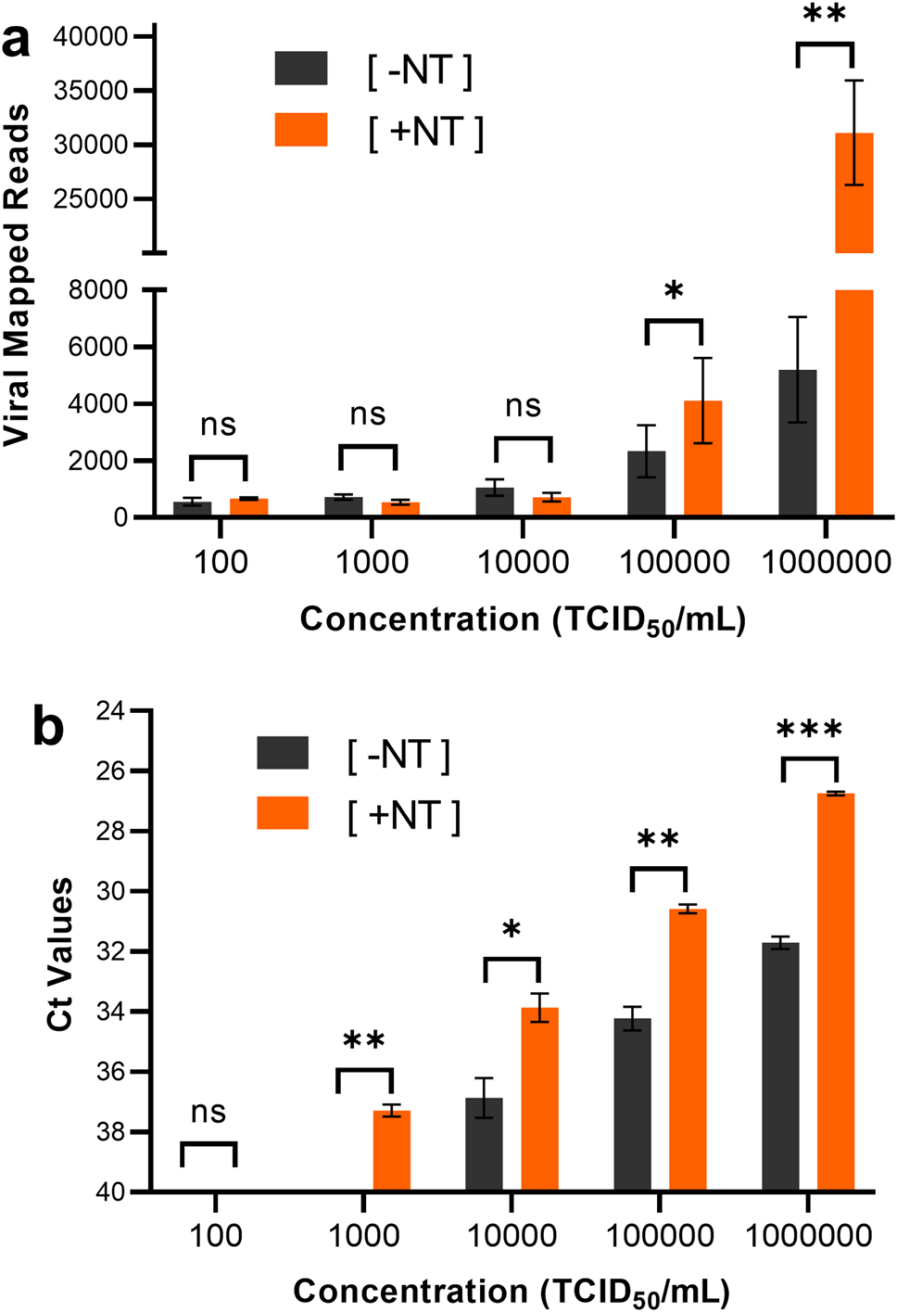
Magnetic Hydrogel Particles (Nanotrap Particle Workflow 1) Improves Sequencing of Contrived SARS-CoV-2 Samples. Heat inactivated SARS-CoV-2 was spiked into VTM at 10^2^, 10^3^, 10^4^, 10^5^, and 10^6^ TCID_50_/mL, and samples were processed using Nanotrap Particle Workflow 1 [+ NT] or the Rneasy Kit alone [− NT]; n = 3 for each process. Samples then underwent sequencing on a ONT MinION R.9 flow cell (**a**) or RT-PCR (**b**). [+ NT] were compared to [− NT] by paired *t*-test in order to assess significance of increased viral detection. *p < 0.05, **p < 0.01, ***p < 0.001.

We developed a method utilizing magnetic hydrogel (Nanotrap particles) to capture and concentrate SARS-CoV-2 whole virions followed by a column-based RNA extraction, examining the Nanotrap particles’ ability to improve nanopore sequencing of SARS-CoV-2. To that end, contrived VTM samples of heat-inactivated SARS-CoV-2 were processed with Nanotrap Particle Workflow 1 using the QIAGEN RNeasy MinElute Cleanup Kit for the viral RNA extraction ([+ NT]) or without Nanotrap particles using the Rneasy kit alone ([− NT]). The extracted RNA samples were prepared for sequencing using the ARTIC nCoV-2019 sequencing protocol and run on the ONT Mk1C sequencer as described in the method section. Extracted viral RNA was also analyzed using the IDT 2019 nCoV CDC EUA RT-PCR Kit to identify a potential correlation between the two assays and confirm the presence of SARS-Cov-2. As shown in Fig. 1A, Nanotrap particles improved sequencing results at multiple concentrations when compared to the workflow without Nanotrap particles. A 6.0 × improvement in SARS-CoV-2 viral mapped reads was observed at 10^6^ TCID_50_/mL and a 2.0 × improvement was seen at 10^5^ TCID_50_/mL. Statistical analysis showed improvements were significant with *p*-values of < 0.05 for both concentrations of virus. No significant improvement was seen between the [+ NT] and [− NT] samples below 10^5^ TCID_50_/mL. When RT-PCR was performed, Nanotrap particles improved viral recovery by 2 PCR cycle thresholds (Cts) across the first four serial dilutions (Fig. 1B). Paired *t*-tests confirmed significant improvement for the same four concentrations.

### Magnetic hydrogel particles (nanotrap particles) improve ONT sequencing results for contrived SARS-CoV-2 VTM samples using a magnetic-bead-based RNA extraction kit

Column RNA extractions are typically used in low sample-throughput, high-complexity laboratory benchtop environments. Given these limitations, we assessed the magnetic hydrogel particles’ (Nanotrap particles’) ability to improve an RNA extraction method based on magnetic beads. Nanotrap Particle Workflow 2 was tested in a similar manner to Workflow 1: contrived VTM samples with SARS-CoV-2 were processed with ([+ NT]) or without ([− NT]) Nanotrap particles. The [− NT] sample was processed using the MagMAX Microbiome Ultra Nucleic Acid Isolation Kit alone. Following RNA extraction, samples were then prepared for sequencing using the ARTIC nCoV-2019 Sequencing protocol, run on the ONT Mk1C Sequencing Platform. Processed RNA samples were also analyzed by RT-PCR using the IDT 2019 nCoV CDC EUA RT-PCR Kit to confirm the presence of virus.

**Figure 2:**
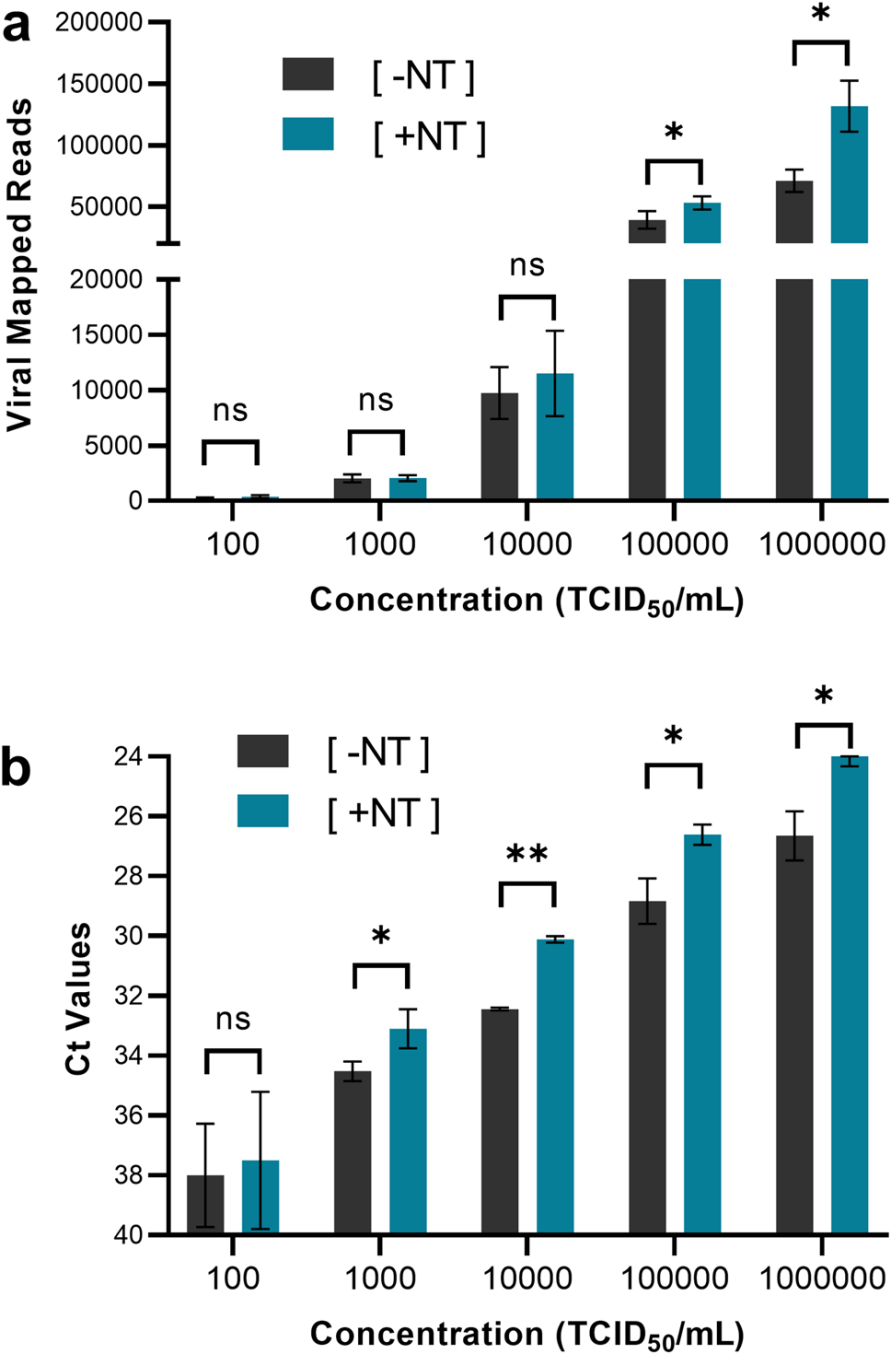
Magnetic Hydrogel Particles (Nanotrap Particle Workflow 2) Improves Sequencing of Contrived SARS-CoV-2 Samples. Heat inactivated SARS-CoV-2 was spiked into VTM at 10^2^, 10^3^, 10^4^, 10^5^, and 10^6^ TCID_50_/mL and samples were processed using Nanotrap Particle Workflow 2 [+ NT] or the MagMAX Kit alone [− NT]; n = 3 for each process. Samples then underwent sequencing on a ONT MinION R.9 flow cell (**a**) or RT-PCR (**b**). [+ NT] were compared to [− NT] by paired *t*-test in order to assess significance of increased viral detection. *p < 0.05, **p < 0.01.

Nanotrap particles improved sequencing results at multiple concentrations when compared to the [− NT] workflow (Fig. 2A). A 1.9 × improvement in SARS-CoV-2 viral mapped reads was observed at 10^6^ TCID_50_/mL and a 1.4 × improvement was seen at 10^5^ TCID_50_/mL. Statistical analysis confirmed significance with *p* values of < 0.05 calculated for both results. Additionally, Nanotrap particles improved SARS-CoV-2 detection in RT-PCR, providing an average 1.5 Ct improvement at viral titers above 10^2^ TCID_50_/mL (Fig. 2B).

### Magnetic hydrogel particles (nanotrap particles) improve ONT sequencing results for diagnostic remnant SARS-CoV-2 VTM samples using a column-based RNA extraction method

**Figure 3:**
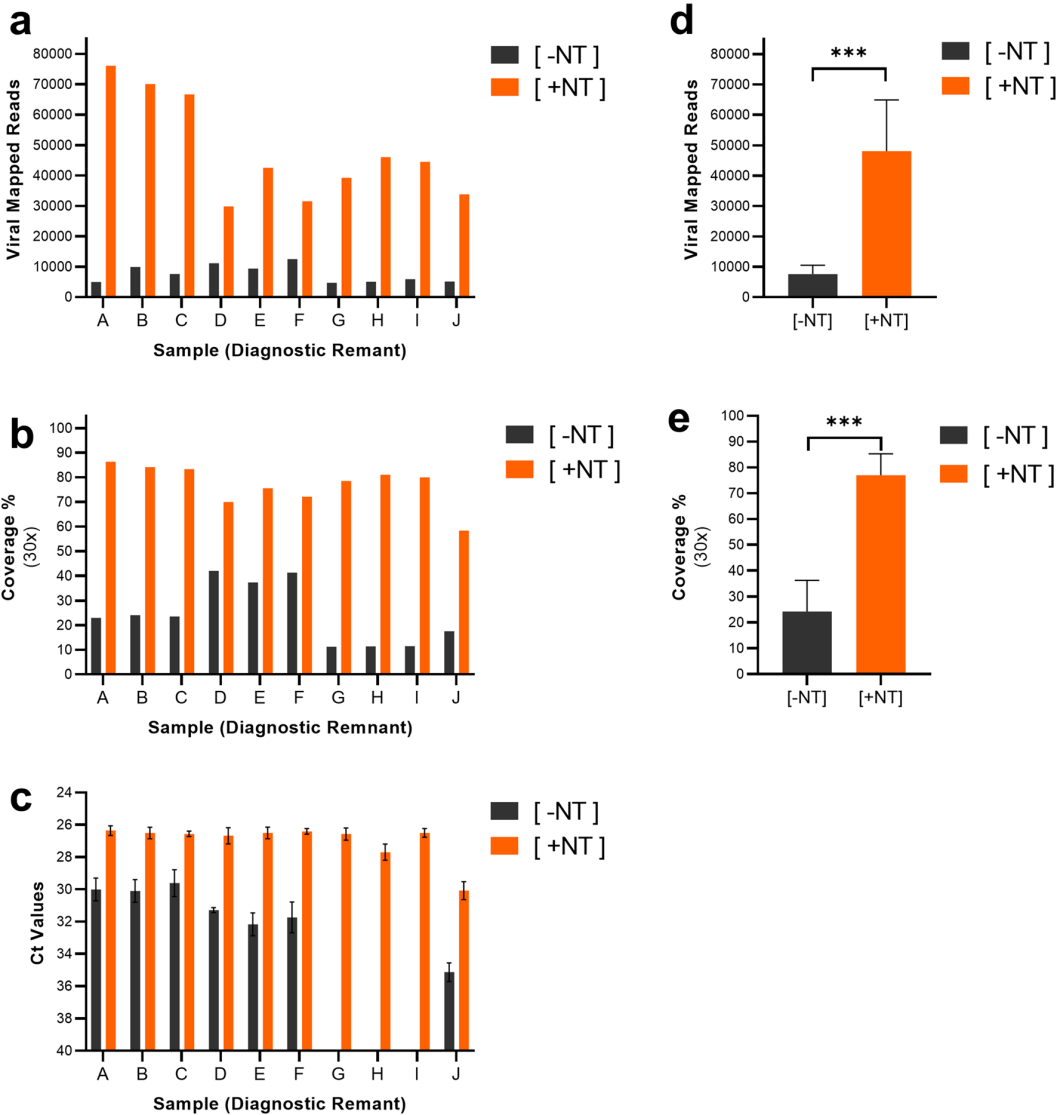
Magnetic Hydrogel Particles (Nanotrap Particle Workflow 1) Improves Sequencing of Diagnostic Remnant SARS-CoV-2 Samples. 10 SARS-CoV-2 positive diagnostic remnant samples were processed using Nanotrap Particle Workflow 1 [+ NT] or the RNEasy Kit alone [− NT]. Samples then underwent sequencing on a ONT MinION R.9 flow cell and were analyzed by Viral Mapped Reads to SARS-CoV-2 (**a**), Viral Genome Coverage at 30 × depth (**b**), or RT-PCR (**c**). [+ NT] were compared to [− NT] by paired *t*-test in order to assess significance of increased viral detection (**d**),(**e**). ***p < 0.001.

Contrived VTM samples are useful for evaluating methods in a pristine environment, but they are not necessarily indicative of how a method will work with clinical samples. Thus, we evaluated the magnetic hydrogel particles (Nanotrap particle) workflows using diagnostic remnant clinical swab VTM samples. We utilized ten diagnostic remnant VTM specimens with reported RT-PCR test cycle thresholds ranging from 24 to 35 (as reported by the specimen supplier). The same processing and sequencing workflow established with contrived samples was used for these diagnostic remnant samples. To better assess the impact of the Nanotrap particle workflow on sequencing results, we quantified both total viral mapped reads and the associated percent genome coverage at 30 × depth of the processed samples. Results in Fig. 3A, which were generated using Nanotrap Particle Workflow 1[+ NT], show that Nanotrap particles improved the sequencing results of 100% of the diagnostic remnant samples (n = 10). Compared to the workflow without Nanotrap particles[− NT], the use of Nanotrap Particle Workflow 1 resulted in an average 7 × improvement in total viral mapped reads across all diagnostic remnant samples. These viral mapped read improvements resulted in an average viral genome coverage increase of 52% over samples processed without Nanotrap particles (Fig. 3B). A paired *t*-test across all 10 samples shows the increases are statistically significant for both the viral mapped reads and coverage percent (Fig. 3D, Fig. 3E). In Fig. 3C, RT-PCR confirmed the presence of SARS-CoV-2 for all 10 samples, also resulting in an average improvement of 4 Ct over [− NT] samples. It is worth noting that 3 samples were below the detection limit of the RT-PCR assay when processed without Nanotrap particles, but all 10 samples had detectable RNA when the Nanotrap particles were used in sample processing.

### Magnetic hydrogel particles (nanotrap particles) improves ONT sequencing results for diagnostic remnant SARS-CoV-2 VTM samples using a magnetic-bead-based RNA extraction kit on a kingfisher automation platform

**Figure 4:**
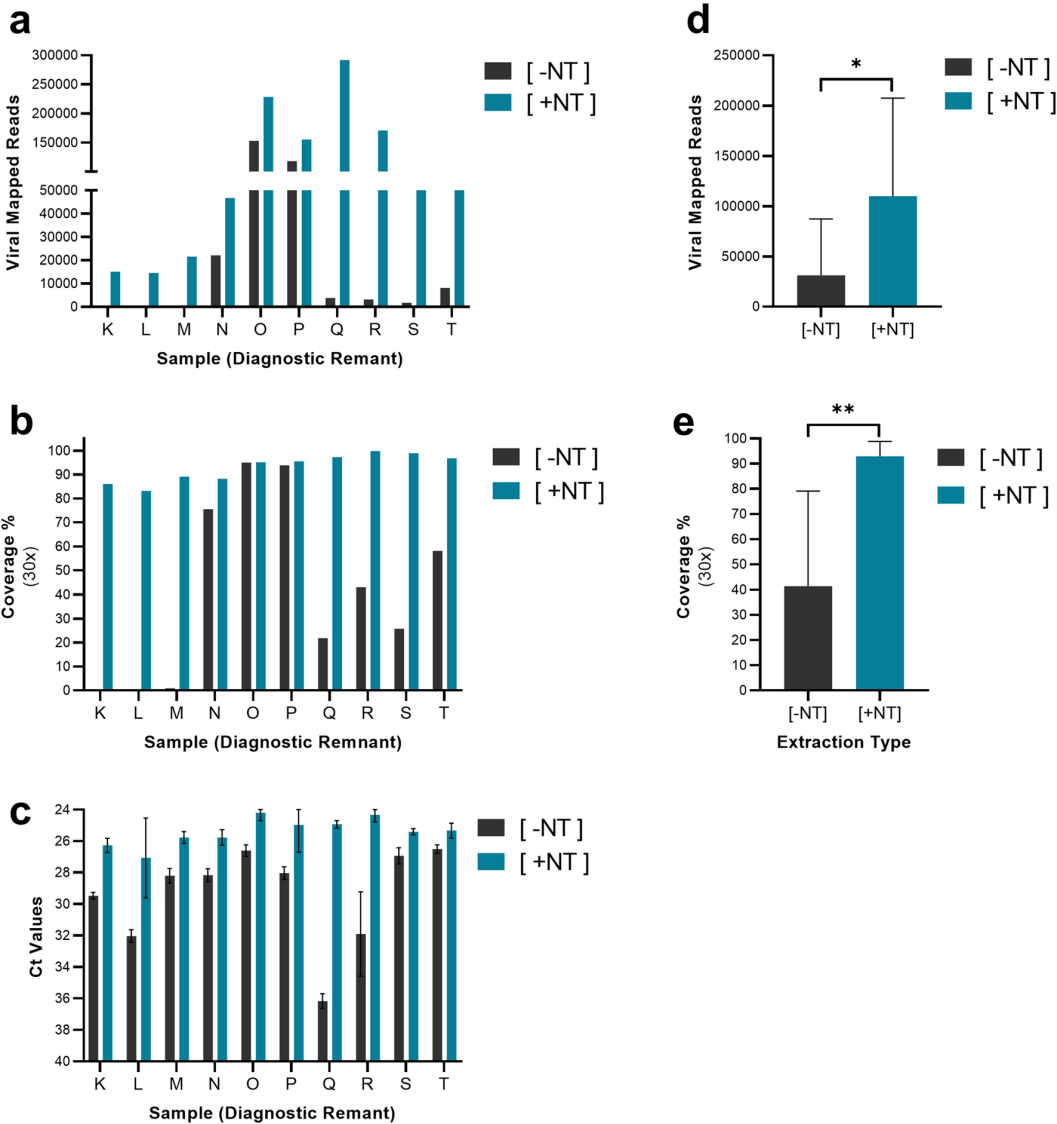
Magnetic Hydrogel Particles (Nanotrap Particle Workflow 3) Improves Sequencing of Diagnostic Remnant SARS-CoV-2 Samples. 10 SARS-CoV-2 positive diagnostic remnant samples were processed using Nanotrap Particle Workflow 3 [+ NT] or the MagMAX kit alone [− NT]. Samples then underwent sequencing on a ONT MinION R.9 flow cell and were analyzed by Viral Mapped Reads to SARS-CoV-2 (**a**), Viral Genome Coverage at 30 × depth (**b**), or RT-PCR (**c**). [+ NT] were compared to [− NT] by paired *t*-test in order to assess significance of increased viral detection (**d**), (**e**). *p < 0.05, **p < 0.01.

One of the advantages of magnetic particle-based sample processing is that the method can be readily automated. To that end, we developed an automated version of magnetic hydrogel particle workflow (Nanotrap Particle Workflow 3) to be used on the KingFisher automation platform. This method is functionally the same as Nanotrap Particle Workflow 2, utilizing the same reagents and extraction steps, benefitting from the same performance characteristics. We compared this automated Nanotrap Particle Workflow 3 method ([+ NT]) to a method without Nanotrap particles using ten additional SARS-CoV-2 positive diagnostic remnant samples ([− NT]), once again examining the sequencing and RT-PCR output of the two methods. We observed that the Nanotrap particle processing significantly improved sequencing results for 7 of 10 samples, resulting in an average improvement of 42 × in total viral mapped reads (Fig. 4A). This corresponded to an average 51% increase in viral genome coverage relative to [− NT] samples (Fig. 4B). Paired t-tests confirmed that Nanotrap particles significantly improved both viral mapped reads (Fig. 4D) and genome coverage (Fig. 4E). As with the previous set of diagnostic remnant samples, RT-PCR confirmed the presence of SARS-CoV-2 for all 10 samples. The [+ NT] automated process improved RT-PCR results as well, resulting in an average 3.7 Ct improvement shown in Fig. 4C.

### Magnetic hydrogel particle (nanotrap particle) enrichment improves bioinformatics analysis of SARS-CoV-2

**Figure 5:**
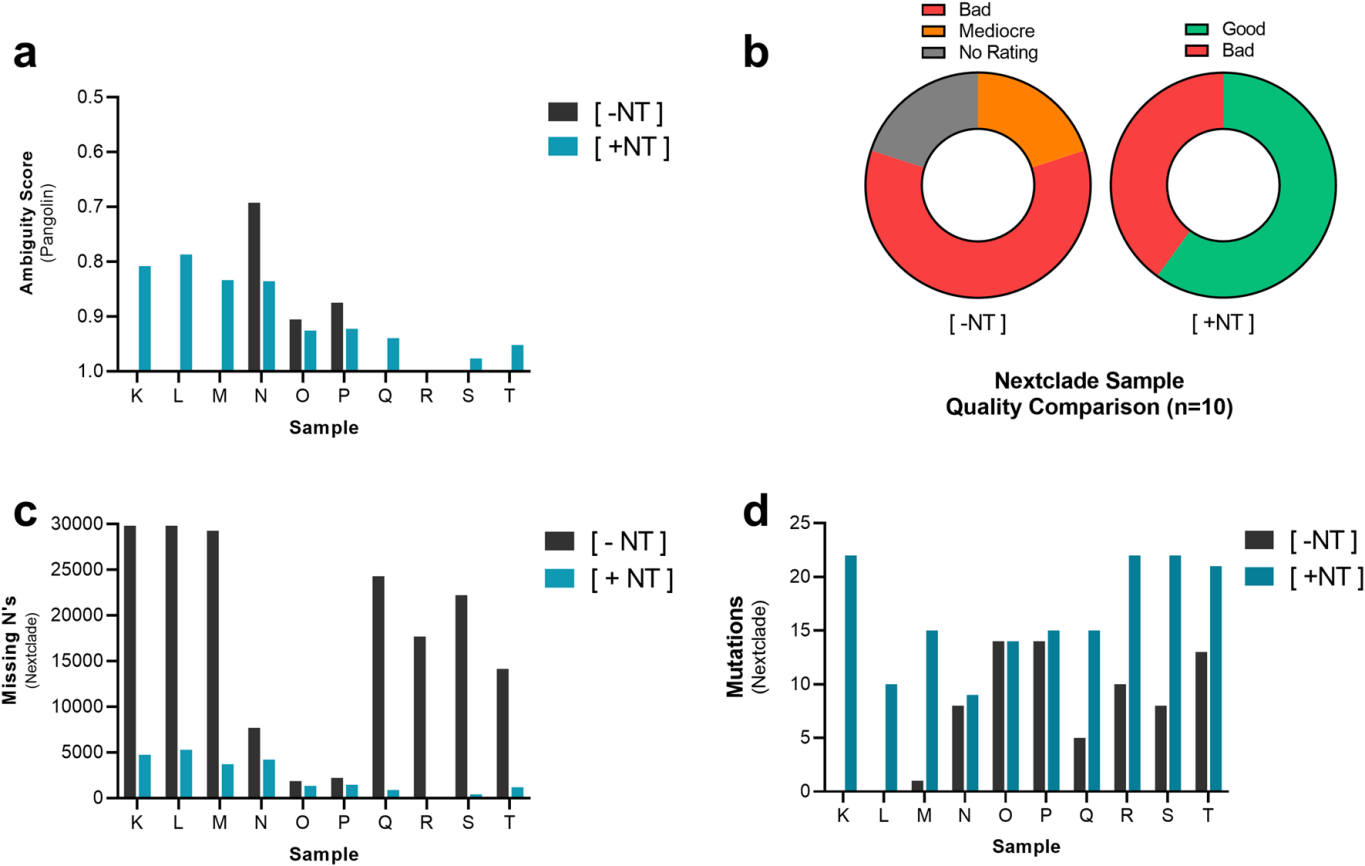
Magnetic Hydrogel Particles(Nanotrap Particles) Improve Bioinformatics Tools when analyzing SARS-CoV-2 Samples. 10 SARS-CoV-2 positive diagnostic remnant samples were processed using Nanotrap Particle Workflow 3 [+ NT] or the MagMAX kit alone [− NT]. Samples underwent sequencing on a ONT MinION R.9 flow cell and were analyzed by Pangolin **(a)**, and Nextclade, which assessed: QC score comparison of all 10 samples **(b)**, total genome locations unable to be characterized per sample (missing n’s, less = better) **(c)**, total mutations detected per sample **(d)**.

After processing and sequencing ten SARS-CoV-2 positive diagnostic remnant samples with Nanotrap Particle Workflow 3, we further assessed how the improvement in viral mapped reads and genome percent coverage would affect downstream SARS-CoV-2 analysis. Specifically, we used the EPI2ME labs bioinformatics tool “ARTIC SARS-CoV-2 Workflow” to perform Pangolin and Nextclade analysis to further characterize the potential improvement gained by magnetic hydrogel particle (Nanotrap particle) enrichment. These are widely used SARS-CoV-2 bioinformatics lineage tools to track and analyze SARS-CoV-2 variants that produce easily understood qualitative metrics for characterizing and comparing sequencing data. Briefly summarized, Pangolin is a SARS-CoV-2 lineage assignment pipeline that starts by aligning an input sequence to the original wild type Wuhan sequence. The tool then compares any changes detected in the aligned coding regions against a database of known variants to generate a lineage report that outputs the closest known variant. Finally, an “ambiguity score” is generated that indicates the likelihood that the lineage report is correct. Ranging from 0–1, the lower the score, the lower the “ambiguity”, which equates to a greater confidence level that the analysis is correct. Similar to Pangolin, Nextclade is a multi-tiered alignment tool that compares an input sequence against the Wuhan sequence identifying mutations that best match previously detected variants, resulting in: a lineage report, mutation count, an alignment map indicating genome coverage, and a general QC metric of the data being analyzed. We utilized Pangolin to generate a lineage report and ambiguity score for both [+ NT] and [− NT] processed samples. For [− NT] samples, Pangolin was only able to generate a lineage report for 3 of 10 samples with an average ambiguity score of 0.824. In the presence of Nanotrap particles [+ NT], however, Pangolin was able to characterize all 10 samples, producing an average ambiguity score of 0.898, indicating that Nanotrap particles were able to improve the software’s ability to characterize samples (Fig. 5A).

Nextclade analysis revealed a similar trend with the use of Nanotrap particles enabling more samples to be characterized at a higher quality rating. Nextclade uses a three tier QC system: “Good”, “Mediocre”, and “Bad”. The QC tier indicates how much of the data provided was able to be characterized by Nextclade to accurately assess the viral genome and determine an accurate classification. When the sample provides insufficient data for any classification to be assigned, samples receive “No Rating”. Here, ten of ten samples processed with Nanotrap particles were characterized, with 6 of 10 samples receiving a QC rating of “good” and 4 of 10 samples receiving a QC rating of “bad”. For [− NT] samples, 2 of 10 samples received a QC rating of “mediocre” and 6 of 10 samples received a QC rating of “bad”. The remaining 2 samples were unable to be characterized and did not receive a rating, indicating that Nanotrap particles improved the software’s ability to characterize samples, enabling previously “Bad” or “Mediocre” samples to become “Good” samples (Fig. 5B). Furthermore, Nanotrap particle processing improved Nextclade analysis by decreasing the amount of missing bases by an average of 15,556 “n”s, or the number of viral genome locations unable to be characterized by Nextclade (Fig. 5C). The “missing n’s” are viral genome blind spots that cannot be used to determine if a mutation exists at that location, these “missing n’s” also contribute to the Nextclade quality rating. The fewer “missing n’s”, the more accurate and useful the Nextclade tool becomes allowing for the analysis of a greater portion of the SARS-CoV-2 genome. For Nanotrap particle processed samples, Nextclade detected an average of 9.2 more mutations per sample compared to samples processed without Nanotrap particles (Fig. 5D). Overall, these results further demonstrate that Nanotrap particle viral enrichment allows for better analysis of samples using common bioinformatics tools.

### Magnetic hydrogel particles (nanotrap particles) improve ONT sequencing results for multiple respiratory viruses

**Figure 6:**
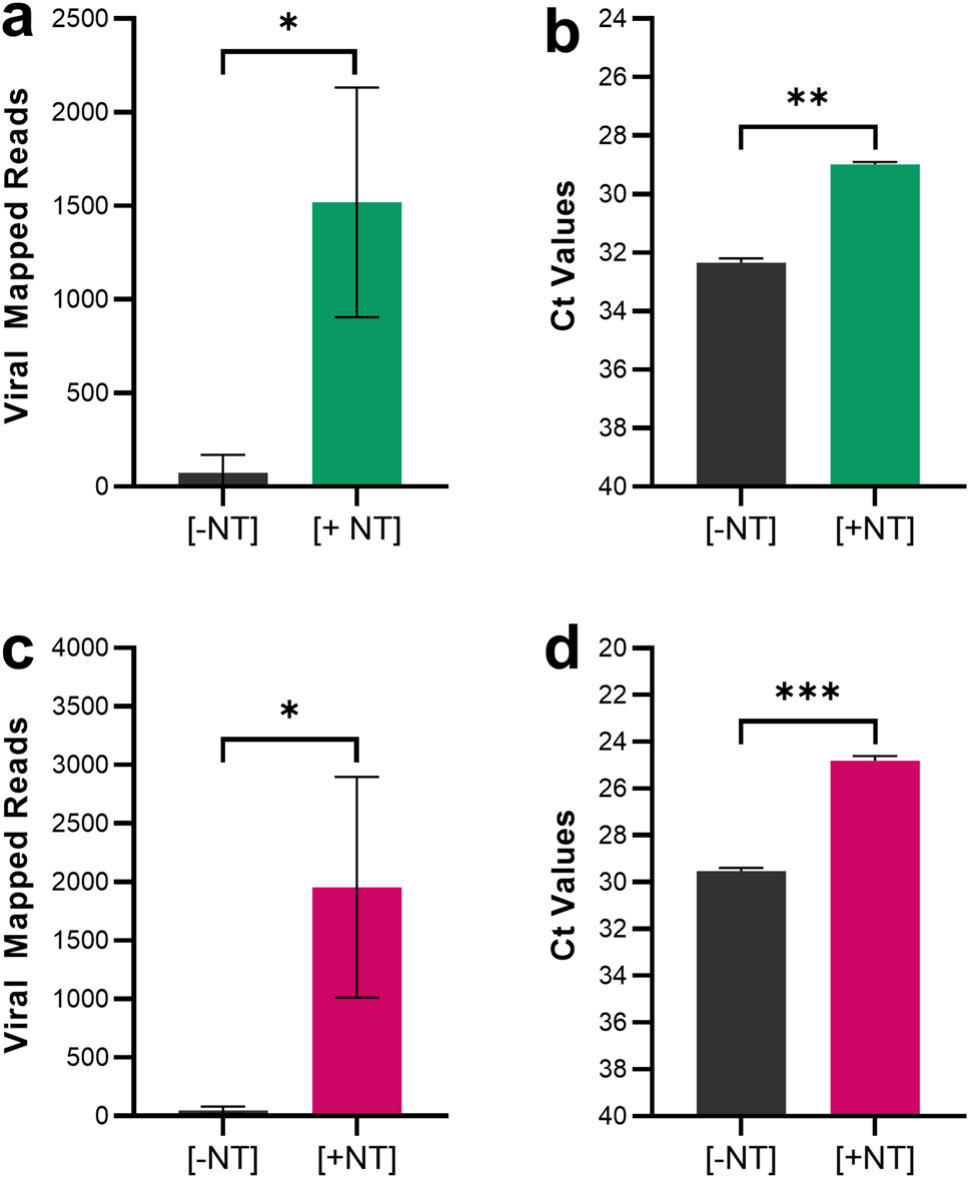
Magnetic Hydrogel Particles (Nanotrap Particles) Improve Sequencing of Multiple Respiratory Viruses. Heat-inactivated influenza A (H1N1) (**a**, **b**) or RSV (**c, d**) was spiked into VTM at 10^6^ TCID_50_/mL and samples were processed using Nanotrap Particle Workflow 1 [+ NT] or the Qiagen RNEasy Kit alone [− NT]; n = 3 for both processes. Samples then underwent sequencing on a ONT MinION R.9 flow cell or RT-PCR. [+ NT] were compared to [− NT] by paired *t*-test in order to assess significance of increased viral detection. *p < 0.05, **p < 0.01 , ***p < 0.001.

Ideally, viral concentration technologies should allow for the concentration of multiple viruses, not just SARS-CoV-2. As prior studies have demonstrated, magnetic hydrogel particles (Nanotrap particles) capture a variety of respiratory viruses; we briefly investigated whether the Nanotrap particles also could be used to improve sequencing of influenza A (H1N1) and respiratory syncytial virus (RSV)^18–20^. Neat VTM was spiked separately at 10^6^ TCID_50_/mL with either inactivated influenza A or RSV. Using the previously established column based RNA extraction protocol, viral contrived samples were processed with Nanotrap Particle Workflow 1[+ NT] and results were compared against samples processed without Nanotrap particles[− NT]. The resulting RNA eluates were then prepared for sequencing using a modified version of the ARTIC Library Prep protocol using primers specific to influenza A and RSV. Samples were run on the ONT Mk1C. Results in Fig. 6A and Fig. 6C demonstrate that Nanotrap particles improve nanopore sequencing results for contrived influenza A and RSV samples, respectively. Nanotrap particles improved viral mapped reads by 4 × for both influenza A and RSV compared to samples processed without Nanotrap particles. Additionally, the use of Nanotrap particles in sample processing improved the detection of viral RNA as measured by RT-PCR for influenza A and RSV, respectively (Fig. 6B, Fig. 6D). Paired *t*-test confirmed the Nanotrap particle-driven improvement was statistically significant with calculated *p* values < 0.05. These results indicate that Nanotrap particles can be used to identify and improve sequencing results of multiple viruses in VTM samples.

## Discussion

Recent advancements in next-generation sequencing and post processing amplification techniques have decreased the viral titers required for successful sequencing runs and accurate mutation detection^27,28^. While these advancements have generally improved the sensitivity of sequencing applications, for certain sequencing platforms there are still a significant portion of clinically relevant viral samples that cannot be sequenced due to low viral titers, samples in which there are insufficient nucleic acid molecules for bioinformatics tools to cover the entire reference genome while confidently distinguishing biological variation from error. More specifically, sequencing platforms “basecall”, or create readouts of the nucleotide fragments from the raw signals generated by the sequencer from the processed RNA samples. These basecalled fragments are compared and mapped against a database to identify the genomic taxonomy of the fragment. During this mapping process, differences will propagate between the analyzed sample and reference genome. These differences in readouts can be due either to real genetic mutations or to erroneous basecalling, the latter of which could be caused by either the sequencer itself or by insufficient nucleic acid material. Increasing the number of basecalled fragments clarifies this problematic overlap with greater read depths, increasing the confidence that detected variations are biological and not an artifact^11–15^.

The Oxford Nanopore MinION sequencing platform is a compact, low complexity third-generation sequencing technology that has significantly reduced the upfront cost typically associated with sequencing. Although this technology has many appealing advantages, the usable clinical sample pool is restricted to higher titer samples due to sensitivity and accuracy limitations^29–31^. We saw this limitation as an opportunity to examine potential enrichment strategies to enhance the amount of nucleic acid material being sequenced, increasing the available pool of clinical samples. To that end, we applied a magnetic hydrogel particle based (Nanotrap particle) front-end virus capture and concentration method to both contrived VTM and diagnostic remnant samples.

Magnetic Hydrogel Particles (Nanotrap particles) significantly improved sequencing results by capturing and concentrating SARS-CoV-2 from contrived samples, improving the output of two standard RNA extraction methods. Furthermore, we identified a general working concentration range of SARS-CoV-2 in which Nanotrap particles were shown to significantly increase the viral mapped reads of the ONT Mk1C sequencing platform. Sequencing and RT-PCR improvements were seen for both Nanotrap Particle Workflows 1 and 2. Relative to the results delivered by the RNA extraction kits without Nanotrap particle pre-processing, both workflows significantly improved total viral mapped reads of SARS-Cov-2 at multiple concentrations. Of the two workflows, greater improvements were seen with the column-based Nanotrap Particle Workflow 1 over its comparator. This workflow employed a larger sample volume, allowing for a more significant amount of enrichment relative to the comparator.

For the magnetic bead-based RNA extraction kit approach, viral mapped reads were generally higher across all concentrations for samples processed with and without Nanotrap particles, relative to the column-based RNA extractions. This suggests greater RNA extraction efficiency of the magnetic bead extraction kit, binding and eluting a higher percentage of RNA. RT-PCR results supported this theory; we observed that the magnetic bead approach allowed detection of SARS-CoV-2 at a tenfold lower concentration than the column-based approach. Viral mapped reads were higher for Nanotrap Particle Workflow 2 vis-a-vis Nanotrap Particle Workflow 1, although the concentration range for which Nanotrap particles significantly improved the number of reads was similar for both workflows, improving sequencing results for the higher concentration samples. While these higher concentrations are typically less problematic for traditional sequencing without pre-enrichment, improving high titer samples can still be useful as sequencing depth can be more quickly reached, thus reducing the time required for successfully sequencing and analyzing these sample types. RT-PCR results showed Nanotrap particle enrichment was efficacious for lower concentration samples for both workflows, suggesting that on an alternative sequencing platform with greater overall sensitivity, Nanotrap particles could also improve sequencing of these lower titer virus samples.

Additionally, results indicate that Nanotrap particle workflows improve sequencing and RT-PCR results of clinically relevant diagnostic remnant samples as compared to the workflows without Nanotrap particles. It appears that Nanotrap particles enhance remnant diagnostic sample sequencing results more significantly than contrived VTM samples. The samples processed in this study were chosen to represent a clinically relevant cohort, with Ct values ranging from 24–35. There did not appear to be a close correlation between Ct values and Nanotrap particle enrichment utility. Interestingly, most diagnostic remnant samples benefitted from Nanotrap particle enrichment regardless of viral concentration. This enhancement is even more compelling when considering the theoretical enrichment resulting from the difference in sample input volume. For Workflow 1 and 3 we expected an improvement of 10 × or 2.5 × relative to the samples processed without Nanotrap particles. Yet, this was far exceeded as we observed upwards of 40 × enrichment in the viral mapped reads of the diagnostic remnant samples. We postulate that VTM collected from humans typically contains greater biological debris, and as a result, the workflows without Nanotrap particles are more likely to be impacted by inhibition while the Nanotrap particle pre-processing reduces this detrimental material through additional sample clean-up. It is possible that the Nanotrap particle architecture enables the capture of the viral material of interest while reducing host cell debris and other contaminating material. The sequencing library preparation workflow assessed here relied on a polymerase-based amplification step which could be negatively impacted by human cellular material. Cleaning up background material while capturing and concentrating viral material would allow the Nanotrap workflow to improve this amplification step even further, thus generating greater total viral mapped reads for diagnostic remnant VTM samples. Additionally, this enrichment was observed for samples with lower Ct’s values, samples which would typically meet the qPCR criteria that dictates a “useful” sample for sequencing but still struggled to generate useful sequencing results in practice. This may indicate that the magnetic hydrogel workflow could be useful for not just low titer samples but also for clinical samples with higher concentrations that fail sequencing.

Nanotrap particles significantly improved viral mapped reads of a large majority of the diagnostic remnant samples for both workflows. As a result, viral genome coverage also increased for a majority of diagnostic remnant samples, increasing by 80% in certain diagnostic remnant samples. These data suggest Nanotrap particles could significantly increase the fraction of samples that could be used for sequencing, though a larger sample set should be run to confirm this. It is worth noting that some of the samples where there was not a statistically significant improvement when the Nanotrap particles were utilized, already had relatively complete genome coverage when processed without Nanotrap particles.

To further assess the practical utility of the improvement seen when using the Nanotrap particles, we also analyzed sequencing data using Pangolin and Nextclade, two bioinformatics tools that are commonly used globally to track and analyze SARS-CoV-2 variants^32–34^. These tools were selected based on their ability to detect and classify mutations, their data requirements for successful variant analysis, and their respective qualitative metrics dictating information quality. This provided a grading system that we used to better determine how impactful Nanotrap particle enrichment is for sequencing data. We observed that for a majority of the diagnostic remnant samples tested, the Nanotrap particle process enabled these bioinformatics tools to work more effectively and, for certain low-titer samples, allowed both Pangolin and Nextclade to complete a successful analysis that would have otherwise failed due to insufficient data. Nextclade was also able to better map the entire SARS-CoV-2 genome for Nanotrap samples, identifying more total mutations per sample relative to the [− NT] control. This suggests that Nanotrap particles potentially allow for researchers to more effectively track and detect variants that would go undetected without the enhanced sensitivity provided by the Nanotrap particle process.

In order for sequencing to become more useful in public health settings, sample throughput and workflow considerations must be addressed. Automated systems, such as the KingFisher automation platform, are readily scalable and already used as a processing tool in clinical laboratories^35^. Nanotrap particles improved the sequencing results of ten positive diagnostic remnant samples when processed using Nanotrap Workflow 3, demonstrating utility in a high throughput automated system. It is worth noting that this automated method would enable the processing of 96 samples in 1 h, which is significantly faster and far more user friendly than the manual-column extraction method, making this an attractive proposition for medium- to high-throughput laboratories.

In addition to SARS-CoV-2, Nanotrap particles have demonstrated use in capturing a broad range of viruses, including respiratory pathogens^17–21^. However, to date, no viral sequencing data has ever been published when using a Nanotrap particle workflow. Here, we confirmed previous reports showing Nanotrap particles can also capture and concentrate influenza A and RSV, two common respiratory viruses. We further demonstrated that our Nanotrap particle workflow is compatible with sequencing of multiple respiratory pathogens, increasing viral mapped reads of both viruses. This suggests that Nanotrap particle workflows can be used for the improvement of broad-scale viral detection by sequencing.

This study contains certain limitations, beginning with the number of samples assessed. A sufficient number of replicates were tested to determine a positive improvement provided by the Nanotrap particle process when sequencing VTM samples, but more samples should be run to better and more accurately quantify the fold-enrichment the workflow can provide. We also did not directly assess the Nanotrap particles’ ability to improve sequencing of different SARS-CoV-2 variants. However, given the general viral capture nature of the Nanotrap particles, we expect that sequencing improvements seen with the wild-type SARS-CoV-2 would correspond to improvements across most other SARS-CoV-2 variants. Future experiments could test this hypothesis by assessing Nanotrap particle enrichment of samples with known variants on the basis of increased detection. We could also create a testing pool of diagnostic remnant samples with known variants at lower titers to examine if the Nanotrap particle workflow can increase the number of available clinical samples for sequencing at one time. Additionally, since only contrived influenza A and RSV samples were examined, we cannot definitively assert at this time that the Nanotrap particle process is capable of sequencing these respiratory pathogens in more biologically complex media. We also did not examine what occurs in samples that are co-infected with multiple viruses. This could potentially bias the Nanotrap particles towards a specific virus should that virus have higher affinity for the Nanotrap particles than others. Future experiments should examine how Nanotrap particles behave in a co-infected sample, along with using more clinically relevant diagnostic remnant samples containing influenza A or RSV. There is also room to explore additional applications of this approach to alternative sample types, including oral fluid (which could be used for less invasive viral respiratory testing) and wastewater (which could be used to conduct surveillance of viral respiratory pathogens in communities). Going forward, we plan to address each of these areas so that we can continue examining viral surveillance applications with this versatile enrichment technology.

Overall, this study indicates that magnetic hydrogel particle (Nanotrap particle) enrichment allows for sequencing of challenging clinical samples in VTM using the ONT MinION Sequencer, samples which otherwise may not have been suitable for sequencing. Because our method requires no filtration or centrifugation steps, this approach is compatible with medium- and high-throughput environments, including the KingFisher automation platform. Additionally, a magnetic hydrogel particle (Nanotrap particle) concentration method paired with an ONT sequencing platform allows for more accessible sequencing efforts. Given both technologies’ relatively low cost and portability, this combined system could potentially be deployed in areas with limited laboratory resources and limited access to more traditional sequencing equipment.

## Data Availability

The datasets generated and/or analyzed during the current study are available in the Github repository, https://github.com/Ceres90/NT-Extraction-Data. Reference Genome: Severe acute respiratory syndrome coronavirus 2 isolate Wuhan-Hu-1, complete genome Accession Number: NC_045512.
